# Parameters Governing the Fate of Fracture Fixation With Proximal Femoral Nailing (PFN) for Intertrochanteric Femur Fractures

**DOI:** 10.7759/cureus.40952

**Published:** 2023-06-25

**Authors:** Aluka Sundeep Reddy Kund, Gopi Krishna Boddana, Chandrasekhar Patnala, Ranjith Yalamanchili

**Affiliations:** 1 Department of Orthopaedics, Nizam's Institute of Medical Sciences, Hyderabad, IND; 2 Department of Orthopaedics, All India Institute of Medical Sciences, Bibinagar, Hyderabad, IND

**Keywords:** intertrochanteric translation, hip offset, greater trochanter orthogonal line, parker's ratio, chang’s reduction quality criteria, proximal femoral nail, parameters of pfn, intertrochanteric fractures

## Abstract

Introduction: A high mortality rate is associated with hip fractures in the elderly. This is because their bones are osteoporotic with implants having less hold and there are more co-morbidities associated with the elderly. Osteosynthesis with the proximal femoral nail (PFN) features the advantages of high rotational stability of the head-neck fragment. However, the use of the nail is technically ambitious and is accompanied by some risks of error, which can lead to failure. This study aims to understand the technical difficulties related to PFN and methods to mitigate them and radiological indicators for successful outcomes of PFN.

Methods: Our study aims to analyze the radiological parameters as indicators for the successful outcome of intertrochanteric fractures fixed using PFN and also the factors responsible for intraoperative conversion to dynamic hip screw (DHS). This is a prospective, observational study conducted from January 2020 to December 2020, on all the patients with intertrochanteric fractures who were planned to be treated by PFN and consented to be part of the study group at our institute. This study includes 99 cases of intertrochanteric fractures classified according to AO (Arbeitsgemeinschaft für Osteosynthesefragen)/Association of the Study of Internal Fixation (ASIF) and Evan’s classification systems and followed postoperatively at regular intervals up to one year. Different methods of fracture reduction, intraoperative radiological parameters, and postoperative radiological parameters dictating the fate of PFN along with factors responsible for the intraoperative conversion to DHS were analyzed and discussed.

Results: Out of 99 patients planned for PFN, four were converted to DHS intraoperatively and 15 patients expired within the follow-up period of one year, leaving only 80 patients in the study group. Of them, seven patients (11.4%) had implant-related complications. According to Chang's reduction quality criteria (CRQC), two cases have CRQC 1 (poor reduction), 11 cases have CRQC 2 (acceptable reduction), 39 cases have CRQC 3 (acceptable reduction), and 28 cases have CRQC 4 (excellent reduction).

Conclusions: Though PFN is technically challenging, with proper guidelines and technique, it gives excellent results. Most importantly, a nonvarus reduction, proper nail insertion and accurate placement of lag screws are the crucial factors for a successful outcome. Biomechanically stable reduction, by closed, percutaneous, or open means, is the key to treating unstable intertrochanteric fracture successfully.

## Introduction

The rate of mortality is considerably high in hip fractures among the elderly [1,2]. The recent shift of treatment paradigm from complete union to early mobilisation is a result of a better understanding of fracture biomechanics and the development of the bio-mechanically stable proximal femoral nail (PFN). Although the pitfalls of the dynamic hip screw (DHS) and advantages of PFN have been well documented and knowledge of correct techniques of PFN is continuously increasing, a detailed analysis of intraoperative parameters to convert to DHS and analysis of post-operative radiographic parameters in successful outcomes is still evolving. This study aims to understand the technical difficulties related to PFN and methods to mitigate them and radiological indicators for successful outcomes of PFN. We also analysed the overall results of PFN, causes of implant failure, and factors responsible for the conversion to DHS intraoperatively.

## Materials and methods

All the patients who were admitted in Nizam's Institute of Medical Sciences, Hyderabad, India, for treatment of intertrochanteric fracture and planned for fixation using PFN pre-operatively from January 2020 to December 2020 were included in the study. This prospective observational study was approved by Nizam's Institute of Medical Sciences Institutional Ethics Committee (approval number: EC/NIMS/2566/2020). Written informed consent was taken from all participants. Patients planned for DHS fixation, pathological fractures, and delayed presentation of more than three weeks were excluded from the study. Patients were followed up at regular intervals until one year after the surgery.

For all participants, a clear history was taken followed by thorough clinical and radiological examinations. They were operated under spinal or epidural anaesthesia using a standard prescribed surgical technique of fixation using PFN (INOR Standard PFN (Wadia Group, Mumbai, India) with 8.0 mm lag screw and 6.4 mm antirotation screw with two distal locking screws)

Intraoperative details of methods of reduction including closed, percutaneous or open means were compared to the final radiological outcome. Intraoperative radiological indices like variance, entry point, anterior cortical alignment, length of hip screw, the position of the hip screw (Parker’s ratio [3] in anteroposterior (AP) and lateral), length of de-rotation screw, greater trochanter orthogonal line, and position of the tip of nail dictating good reduction, and factors responsible for difficulty and failure in procedure and intraoperative shift to DHD were analysed.

**Figure 1:**
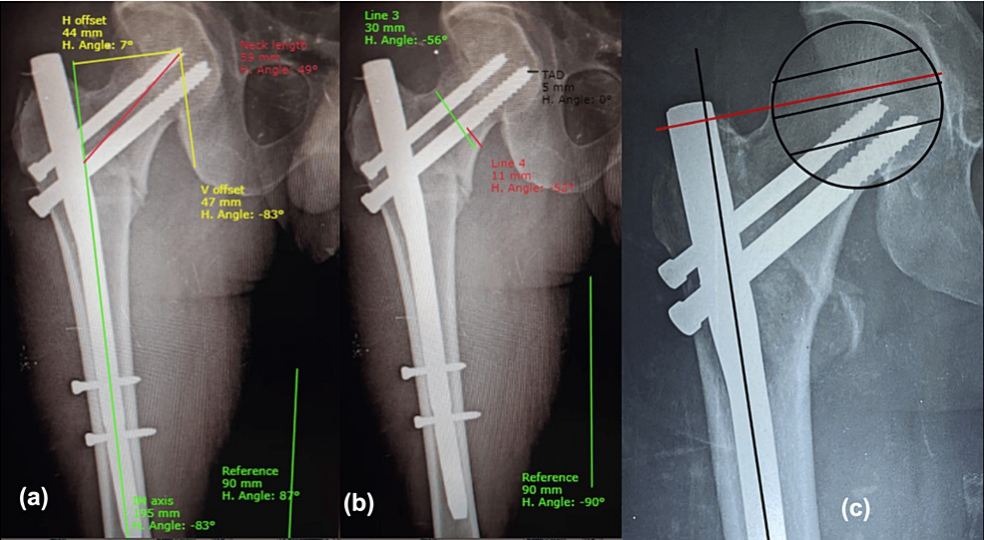
Postoperative analysis of radiographs using Surgimap software* (a) Horizontal offset = 44mm, Vertical offset = 47mm, Neck length = 59mm; (b) Parker's ratio = 11/30=0.34, CalTad = 5mm;  (c) GTOL (red line) passing through second quadrant of femoral head. V: vertical; H: horizontal; CalTAD: calcar referenced tip-apex distance; GTOL: greater trochanter orthogonal line; IM: intramedullary *Nemaris, Inc., New York, United States

Postoperative analysis of radiographs by means of reconstructed hip measurements included neck shaft angle, neck length and horizontal offset, calcar referenced tip-apex distance (CalTAD) [3], Parker’s ratio [3] and Chang’s reduction quality criteria (CRQC) [4] and their correlation with patient’s functional outcome were analysed. All the hip measurements and radiographic analyses were made on AP radiographs of the hip in 15 degrees internal rotation and cross-table lateral view, using Surgimap software (Nemaris, Inc., New York, United States) (Figure 1). On-bed mobilisation is started from postoperative day (POD) 1. Weight-bearing in all cases was started at three to six weeks depending on the quality of reduction, quality of bone, and general condition of the patient. Six months after surgery, all the included patients were followed up and analysed for clinical and functional outcomes by means of the Harris Hip Score questionnaire [5]. Mean and standard deviation (SD) are computed for quantitative variables like CalTAD, horizontal offset, neck length, etc. Frequency and percentage will be calculated for qualitative variables like gender, mode of injury, type of fracture, and functional outcome (excellent to poor). 

## Results

The mean age in the present study was 59.59 ± 14.99 years. The sex distribution of the subjects shows the current study consisted of 42 males and 38 females (1.1:1). Majority of the subjects were in the overweight range (n=47; 58%) followed by healthy (n=25; 31%) and eight subjects (10%) were obese. Fifteen subjects (18.7%) expired within the study period. Most of the expired patients were above the age of 70 years and associated with two or more comorbidities and most of them had unstable trochanteric fractures. Classifying all the subjects based on Singh’s index of osteoporosis [6], 27 subjects (33.7%) were seen to have grade 3 osteoporosis, 33 (41.25%) subjects had grade 4, 17 (21.25) subjects had grade 5, and the remaining three (3.75%) subjects had grade 6. Broadly, 26 cases (32.5%) were stable fractures and 54 cases (67.5%) were unstable fractures. 

Method of reduction 

Among the 26 stable fractures, 22 subjects underwent closed reduction in a sequence of abduction, external rotation (unlocking of fragments), adduction, and internal rotation, and four subjects underwent percutaneous reduction. Among 54 unstable fractures, 24 subjects underwent closed type of reduction, seven subjects underwent open type of reduction, and 23 subjects underwent percutaneous type of reduction. All the stable fractures had acceptable fracture reduction and among those with unstable fractures, 48 subjects had acceptable fracture reduction, six had poor fracture reduction, and four had unacceptable fracture reduction and were converted to DHS intraoperatively (Table 1). 

**Table 1:** Intraoperative analysis of type of reduction, entry point, variance, and quality of reduction

Intraoperative Findings	Stable Fractures	Unstable Fractures	Total
Type of reduction			
Closed	22 (84.6%)	24 (44.4%)	46 (57.5%)
Open	0	07 (13.0%)	07 (8.8%)
Percutaneous	04 (15.4%)	23 (42.6%)	27 (33.8%)
Fracture reduction quality			
Acceptable	26 (100.0%)	47 (88.9%)	73 (92.5%)
Poor	0	03 (3.7%)	03 (2.5%)
Unacceptable	0	04 (7.4%)	04 (5.0%)
Entry point			
Medial to tip	19 (73.1%)	31 (57.4%)	50 (62.5%)
Pyriformis	05 (19.2%)	22 (40.7%)	27 (33.8%)
Tip of Greater trochanter	02 (7.7%)	01 (1.9%)	03 (3.7%)
Total	26 (100.0%)	54 (100.0%)	80 (100.0%)
Variance			
Negative	02 (7.7%)	12 (24.0%)	14 (18.4%)
Positive	06 (23.1%)	12 (24.0%)	18 (23.7%)
Neutral	18 (69.2%)	26 (52.0%)	44 (57.9%)
Total	26 (100.0%)	54 (100.0%)	80 (100.0%)

Entry point

Entry point (in AP view) is medial to greater trochanteric (GT) tip in 19 (73.1%) and 31 (57.4%), through piriformis in five (19.2%) and 22 (40.7%), and through tip of GT in two (7.7%) and one (1.9%) of stable and unstable fracture patterns, respectively (Table 1). 

Variance

In AP radiographs of the hip, positive variance is achieved in six (23.1%) and 12 (24.0%), neutral variance in 18 (69.2%) and 26 (52.0%), and negative variance in two (7.7%) and 12 (24.0%) in stable and unstable fracture patterns, respectively (Table 1). 

Position of screw in head and Parker’s ratio

Sixty-one cases had Parker’s ratio of <0.33 (inferior quadrant) in AP view and <0.33 (posterior quadrant) in lateral view, and 19 cases had a ratio of 0.34-0.66 (central quadrant) in both AP and lateral radiographs. The mean Parker’s ratio for stable and unstable fractures in AP radiographs was 0.27±0.06 and 0.3±0.09, respectively (p value=0.04) and the mean Parker’s ratio for stable and unstable fractures in lateral radiographs was 0.51±0.1 and 0.46±0.09, respectively (p value=0.04) (Table 2). 

**Table 2:** Distribution of subjects according to position of screw in head (AP and lateral) and relation with Parker's ratio and mean Parker's ratio in both stable and unstable fractures AP: anteroposterior; SD: standard deviation; X^2 ^: Chi square value

Position of screw in head (AP)	Parker’s ratio (AP) 0-0.33	Parker’s ratio (AP) 0.34-0.66	Parker’s ratio (AP) 0.67-1	
Centre	03 (4.9%)	15 (79.0%)	0	20 (25.0%)
Inferior	58 (95.1%)	02 (10.5%)	0	57 (71.2%)
Superior	0	02 (10.5%)	0	03 (3.8%)
Total	61 (100.0%)	19 (100.0%)	0	80 (100.0%)
	Χ^2^ value	46.411	p-value	<0.001
Position of screw in head (Lateral)	Parker’s ratio (Lateral) 0-0.33	Parker’s ratio (Lateral) 0.34-0.66	Parker’s ratio (Lateral) 0.67-1	Total
Anterior	02 (3.3%)	0	0	02 (2.5%)
Central	58 (95.1%)	02 (10.5%)	0	60 (75.0%)
Posterior	01 (1.6%)	17 (89.5%)	0	18 (22.5%)
Total	61 (100.0%)	19 (100.0%)	0	80 (100.0%)
	Χ^2^ value	38.040	p-value	<0.001
Parker’s ratio (Mean±SD)	Stable Fractures	Unstable Fractures	Total	p-value
Parker’s ratio AP	0.27±0.061	0.30±0.092	80	0.04
Parker’s ratio lateral	0.51±0.102	0.46±0.091	80	0.044

Length of hip screw and de-rotation screws

After appropriate reduction is achieved either by closed, percutaneous, or open method, the inferior (hip screw) guide wire is passed, followed by the superior (de-rotation screw) guide wire, and reaming of appropriate size is done simultaneously (8 mm for inferior and 6.4 mm for superior). Similarly, screws are put in simultaneously to avoid rotational deformity of proximal and distal fragments and to avoid loss of reduction. The distribution of screws was as follows: 80 mm hip screw in 10 cases (12.5%), 85 mm in 27 (33.8%), 90 mm in 26 cases (32.5%), and 95 mm in 17 cases (21.3%); de-rotation screw of 65 mm in four cases (1.3%), 70 mm in 22 cases (27.5%), 75 mm in 40 cases (50%), 80 mm in 10 cases (12.5%), 85 mm in three (3.8%), and couldn’t be applied in one case (Table 3).

**Table 3:** Distribution of subjects according to length of proximal screws

Type of screws	Frequency (n=80)	Percent
Hip Screws		
80 mm	10	12.5
85 mm	27	33.8
90 mm	26	32.5
95 mm	17	21.3
De-rotation Screws		
65 mm	4	1.3
70 mm	22	27.5
75 mm	40	50.0
80 mm	10	12.5
85 mm	3	3.8
Couldn't be applied	1	1.3

Greater trochanter orthogonal line (GTOL)

The GTOL is a line passing from the tip of the GT, parallel to the horizontal through the femoral head, which is divided into four quadrants from upwards to downwards. It shows the relation between the tip of the GT and the centre of the femoral head. TheGTOL passed through the first quadrant in 14 cases, which were all unstable, and through second quadrant in 66 cases, out of which 26 are stable and 40 are unstable.

Neck shaft angle (NSA)

The mean NSA reconstructed in stable fractures was 130.7±2.73° and in unstable fractures, it was 128.9±3.44° in the immediate postoperative period. The mean NSA at the end of six months in stable fractures was 128.6±2.87° and in unstable fractures it was 124.6±6.69°, i.e. the mean NSA lost at the end of six months was 2.0±1.17° and 3.1±3.97°in stable and unstable fractures, respectively (Table 4).

**Table 4:** Distribution of subjects according to postoperative findings in NSA and neck length NSA: neck shaft angle; SD: standard deviation

Postoperative findings	Stable Fractures (Mean±SD)	Unstable Fractures (Mean±SD)	p-value
Neck-shaft angle (in degrees)			
Uninjured side	131.0±3.14	129.8±3.01	0.136
Immediate postoperative	130.7±2.73	128.9±3.44	0.036
After weight bearing	128.6±2.87	124.6±6.69	0.005
Lost after weight bearing	2.0±1.17	3.1±3.97	0.018
Neck length (in mm)			
Uninjured side	59.2±3.57	57.6±3.64	0.101
Postoperative	56.8±5.05	56.1±4.29	0.496
Lost after weight bearing	2.9±2.38	2.4±1.55	0.261

Neck length

The mean neck length reconstructed in stable fractures is 56.8±5 mm and 56.1±4.29 mm in unstable cases. The mean neck length lost at the end of six months is 2.9±2.38 mm in stable and 2.4±1.55 mm in unstable fractures (Table 4).

Horizontal offset

The mean reduction in horizontal offset among those with stable and unstable fractures was 2.32±1.21 mm and 2.05±1.34 mm, respectively. The mean reduction in horizontal offset among those with acceptable CRQC and excellent CRQC was 2.29±1.33 mm and 1.96±1.23 mm, respectively (Table 5).

**Table 5:** Distribution of subjects according to horizontal offset *Level of significance: 0.05 SD: standard deviation

Pre-op fracture status	Stable Fractures (cm)	Unstable Fractures (cm)	p-value
Horizontal offset (Mean±SD)	2.22±1.215	2.55±1.341	0.041
Fracture reduction quality	Acceptable	Excellent	
Horizontal offset (Mean±SD)	2.29±1.334	1.96±1.232	0.036*

CalTAD

The mean CalTAD of stable and unstable fractures was 19.5±2.58 mm and 20.9±3.4 mm, respectively (p-value 0.08).

CRQC

Out of 26 stable fractures, the quality of reduction was excellent (CRQC score 4) in nine cases and acceptable reduction (CRQC scores 2 and 3) in 17 cases. Out of 54 unstable fractures, there was excellent CRQC in 26 cases, acceptable CRQC in 26 cases, and poor CRQC in two cases (Table 6).

**Table 6:** Distribution of subjects according to fracture reduction quality (CRQC) CRQC: Chang's reduction quality criteria

CRQC	Frequency (n=80)	Percent	Fracture Reduction Quality	Frequency (%)
Score 0	0	0%	Poor	2 (2.5%)
Score 1	2	2.5%		
Score 2	11	13.8%	Acceptable	50 (62.5%)
Score 3	39	48.7%		
Score 4	28	35.0%	Excellent	28 (35.0%)

HHS functional outcome 

The HHS showed excellent outcomes in 48 (60%) cases, good in 29 (36.2%) cases, and fair to poor in three cases (3.8%). The association between preoperative fracture status and functional outcome was found to be statistically significant (p=0.031), meaning the majority of those with poor outcomes had unstable fracture patterns, while most of those with good and excellent outcomes had stable fractures (Table 7).

**Table 7:** Distribution of subjects according to functional outcome (Harris Hip Score) ^a^ AO classification; *Level of significance: 0.05 X^2^: Chi square

Harris hip score		Functional Outcome			Frequency (n=80)		Percent	
<70		Poor			03		3.8%	
70-80		Fair			0		0%	
81-90		Good			29		36.2%	
91-100		Excellent			48		60.0%	
Functional outcome	Χ^2^value		OR	95% CI				p-value
				Lower		Upper		
Preoperative fracture status ^a^	18.250		1.075	1.001		1.167		0.031^*^

Adverse events

Table 8 shows the distribution of subjects according to intraoperative adverse events. Among those with stable fractures, there was difficulty in reducing the fracture while performing the surgery in four subjects but among those with unstable fractures, the procedure was converted into DHS in four subjects, there was difficulty in reducing the fracture while performing the surgery in 27 subjects, and in one subject, there was failure to put in de-rotation screw. In total, the procedure was converted into DHS in four subjects, there was difficulty in reducing the fracture while performing the surgery in 31 subjects, there was a failure in placing de-rotation screw in one subject, and lateral wall collapse/GT split/comminution was seen in seven subjects.

**Table 8:** Distribution of subjects according to adverse events (intraoperative and postoperative) DHS: dynamic hip screw; GT: greater trochanter; X^2^ : Chi square

Intraoperative adverse events	Stable Fractures (n=26)	Unstable Fractures (n=58)	Total (n=84)
Converted to DHS	0	4 (6.9%)	4 (4.8%)
Difficult closed reduction	4 (15.3%)	27 (46.5%)	31 (40%)
Failed de-rotation screw	0	1 (1.7%)	1 (1.2%)
Lateral wall collapse/ GT split/Comminution	1 (3.7%)	6 (10.3%)	7 (8.3)
Zig mismatch	0	0	0
Failure of distal locking	0	0	0
None	21 (81.0%)	20 (34.6%)	41 (48.8%)
χ ^2 ^value=17.468, p-value=0.002			
Postoperative adverse events	Stable Fractures (n=26)	Unstable Fractures (n=54)	Total (n=80)
Abductor lurch	0	11 (20.4%)	11 (13.7%)
Z effect	0	2 (3.7%)	2 (2.5%)
Reverse Z effect	0	0	0
Screw cut	0	2 (3.7%)	2 (2.5%)
Implant removal	0	1 (1.9%)	1 (1.3%)
Lateral thigh pain	1 (3.8%)	11 (20.4%)	12 (15.0%)
None	25 (96.2%)	27 (50.0%)	52 (58.0%)
χ ^2 ^value=14.811, p-value=0.022			

Table 8 also shows the distribution of subjects according to post-operative adverse events. Among those with stable fractures, lateral thigh pain occurred in one but among those with unstable fractures, abductor lurch was seen in 11 subjects, Z effect in two subjects, two subjects had screw cut, lateral thigh pain was there in 11 subjects, and in one subject, the implant was removed. In total, there was abductor lurch in 11 subjects, Z effect in two subjects, two subjects had screw cut, lateral thigh pain occurred in 12 subjects, and in one subject the implant was removed.

## Discussion

All parameters responsible for reduction and postoperative outcomes were analysed. We observed that as we progress from grade 3 to grade 6 osteoporosis, reduction quality improves and mean HHS increases exponentially, which is statistically significant (Table 9); p-value (osteoporosis vs CRQC) = 0.007, p-value (osteoporosis vs HHS) = 0.04, which is in concurrence with the study done by Akan et al. [7], which showed better HHS in grade 4 and 5 osteoporosis than in grade 2 and 3. 

**Table 9:** Singh's index (Grades 1-6) in comparison to CRQC and HHS CRQC: Chang's Reduction Quality Criteria; HHS: Harris Hip Score

	Grade		CRQC (1)	CRQC (2/3)	CRQC (4)	Total cases	HHS (Mean)
Singh's index	3	Count	1	5	20	27	
		%	50%	17.8%	40%	33.75%	75
	4	Count	1	12	20	33	
		%	50%	42.8%	40%	41.2%	77
	5	Count	0	11	6	17	
		%	0.0%	39.2%	12%	21.2%	85
	6	Count	0	0	4	3	
		%	0.0%	0.0%	8%	3.75%	91
Total		Count	2	28	50	80	
		%	100.0%	100.0%	100.0%	100.0%	82 (mean of all cases)
		Chi-Square Value			p-value		
Osteoporosis vs CRQC		14.441			0.007		
Osteoporosis vs HHS		16.44			0.04		

**Figure 2:**
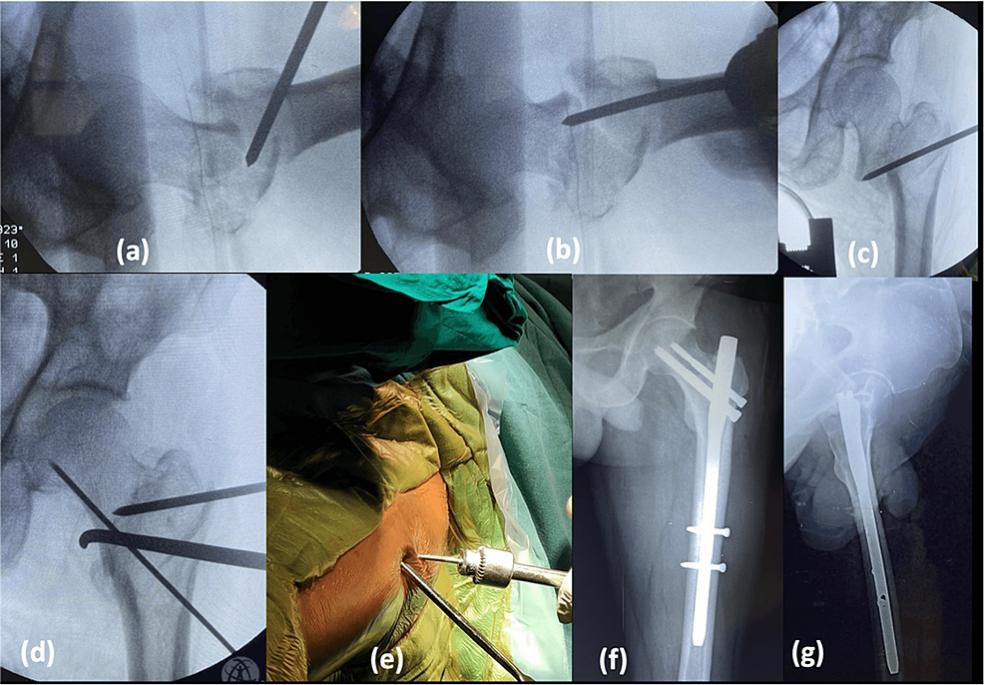
Percutaneous reduction and provisional fixation. (a) Intussusception of proximal medial beak into distal canal; (b) Lifting the sag with K-wire maintaining anterior cortical continuity; (c) Lifting force pushing the medial beak more medial; (d) Bone hook used to align the medial cortical continuity; (e) Clinical picture of percutaneous reduction; (f) Postoperative radiograph, AP; (g) Postoperative radiograph, lateral K-wire: Kirschner wire

In the majority of the stable fractures, reduction was obtained in closed method with traction table (22/26 subjects) and 4/26 needed percutaneous reduction. In unstable fractures, closed method was only successful in 24/54 subjects, and in 30/54 subjects, percutaneous/open methods were used to reduce the fracture prior to taking an entry point. This is because the PFN implant does not ensure fracture reduction; therefore, prior fracture reduction is a must by any means (closed, percutaneous, open). Jain et al. studied the role of provisional fixation of fracture fragments by Steinmann pin in PFN for intertrochanteric fractures and concluded that provisional fixation of fracture by Steinmann pin/K wires (Kirschner wire) after achieving a good reduction is a must for non-varus reduction, inserting nail correctly and accurately placing lag screws [8]. The most common method of percutaneous reduction used in unstable injuries is by intrafocal manipulation with Steinmann pin, by passing at the fracture site and lifting the depressed fragment (either the neck or the shaft) (Figure 2).

Once the anterior cortex is well aligned, percutaneously it is fixed with two K-wires which are very anteriorly placed. Once the fracture is fixed temporarily with K-wires, the hip is adducted without fracture going into the varus; thus, nailing becomes easy.

**Figure 3:**
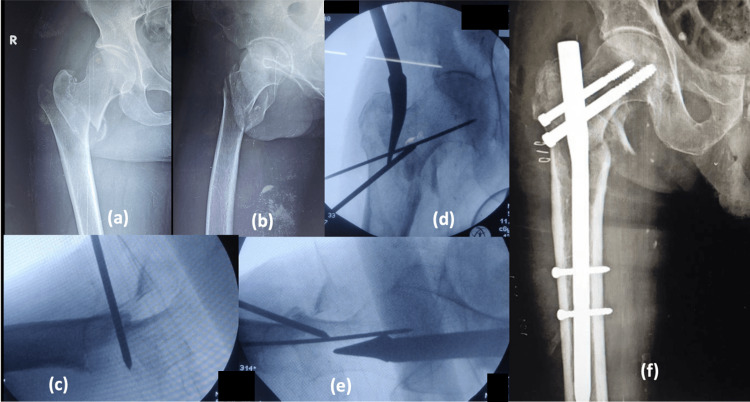
Piriformis entry after percutaneous reduction (a) Unstable trochanteric fracture, AP; (b) Unstable trochanteric fracture, Lateral; (c) Percutaneous reduction, anterior cortical alignment; (d) Piriformis entry, AP; (e) Piriformis entry, Lateral; (f) Postoperative radiograph, AP AP: anteroposterior

The entry point for the insertion of PFN is also an important factor contributing to fracture reduction. In unstable fractures, the entry point needs to be more medialized to get a more cortical grip (more reaming towards the neck) (Figure 3), as there will be GT comminution. If the entry is taken at the tip of GT or medial to GT, with further proximal reaming and nail passage, the final placement of the nail will be more lateral with minimal or no lateral support and finally end in varus and improper screw placement. In our study, there was a significant association between the entry point taken and fracture reduction quality (p =0.004), i.e., there are fewer/no difficulties or complications when the entry point is taken through the medial biased tip of GT and piriformis and higher complication rates/difficulties when the entry point is taken through the tip of GT, which is in concurrence with Shivshankar et al. [9].

The current study showed the position of lag screw in AP, which was central in 20 subjects (25%), inferior in 57 subjects (71.2%) and superior in three subjects (3.8%). As the inferior screw is placed more superiorly (increase in Parker’s ratio) in the head, the reconstructed NSA gradually decreased meaning more varus. A statistically significant association was found between the position of the screw to that of NSA and Parker’s ratio (p-value<0.001) (Table 10). Similar to our study, the quality of reduction and Parker’s ratio demonstrated a significant correlation in univariate analysis with cut out in a study done by Murena et al. [4]. The position of the screw in lateral view was central in 60 subjects (75%), anterior in two (2.5%) subjects, and posterior in six (22.5%) subjects. There was a statistically significant association found between the position of the screw in lateral and Parker’s ratio with a p-value<0.00.

**Table 10:** Analysis of position of Calcar screw versus resultant NSA and significance Χ^2^ value: 55.674; p-value <0.001 AP: anteroposterior; NSA: neck shaft angle; X^2^: Chi square

Position of calcar screw in head (AP)	NSA<122^0 ^(n=6)	NSA=122-128^0 ^(n=29)	NSA >129^0 ^(n=45)	Total (n=80)
Centre	3 (50.0%)	15 (52.4%)	2 (4.4%)	20 (25.0%)
Inferior	0	14 (47.6%)	43 (95.6%)	57 (71.2%)
Superior	3 (50.0%)	0	0	3 (3.8%)

In our study, we noticed the correlation between the screw position and expected NSA as inferior screw placement usually had an NSA of 130 degrees, whereas central screw placement had an NSA of 126 degrees and superior screw placement had an NSA of 120 degrees (Table 11).

**Table 11:** Mean NSA vs position of Calcar screw NSA: neck shaft angle; SD: standard deviation; AP: anteroposterior

Calcar screw position in head	Minimum NSA reconstructed	Maximum NSA reconstructed	Mean NSA reconstructed	SD
Inferior (AP)	125	136	130.73	2.285
Centre (AP)	120	130	126.07	2.921
Superior (AP)	120	120	120.20	2.124

In the study by Yan et al., the screw position was ideal (inferior on AP radiograph and central on lateral radiograph of femoral head) in 48 and 79 in gamma nail (GN) and PFN antirotation (PFNA) groups, respectively, acceptable (central on AP radiograph and central, anterior, or posterior on lateral radiograph of femoral head) in 25 and 19 in GN and PFNA groups, respectively, and poor (superior on AP radiograph and central, anterior, or posterior on lateral radiograph of femoral head) in 23 and 14 in GN and PFNA groups, respectively [10]. Menezes et al. reviewed 155 consecutive patients and reported the position of femoral neck screws was unsatisfactory in 23 patients (15%); this problem was not related to fracture type (17.5% versus 12.9% versus 18.2%) [11]. Jain et al., in their prospective clinical study, reported that in immediate postoperative radiographs, 46 screws were in the centre or inferior position while four were in the superior part of the neck on AP projection [8].

The most commonly used hip screws in our study were 85 mm (27/80; 33.8%) and 90 mm (26/80; 32.5%) followed by 95 mm screws (17/80; 21.3%). The most commonly used de-rotation screws are 70 mm (22/80; 27.5%) and 75 mm (40/80; 50%). We have taken into consideration the difference between both the screws and correlated them with the complications and HHS in the postoperative and follow-up period (Table 12). There is a significant association between the length of the de-rotation screw in comparison to the hip screw and the mechanical complications and clinical outcome (HHS). In our study, we have observed all the screw-related mechanical complications (screw backout and cutout and Z/reverse Z effects) in the cases where the de-rotation screw is less than 10 mm in comparison to the hip screw, and also the HHS being low with differences less than 10 mm. Morihara et al. also opine that the hip pin should be shorter by at least 15 mm, otherwise, it could take the weight and back out or migrate into the joint leading to cut out [12].

**Table 12:** Difference in proximal screw lengths vs screw complications vs HHS HHS: Harris Hip Score

Difference in screw length	Number of cases	Screw-related complications	Mean HHS
5 mm	5	3	70
10 mm	12	2	68
15 mm	42		94
20 mm	21		92

**Figure 4:**
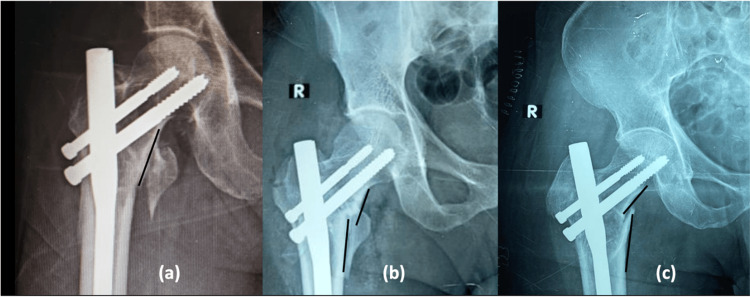
Variance: (a) Neutral, (b) Positive, (c) Negative R: right

Out of the 80 cases included in our series, we observed that negative variance is predominant in the unstable fractures group due to the posteromedial comminution and difficult fracture reduction (Figure 4). Variance in comparison to NSA and functional outcome (HHS) was statistically significant (p-values 0.005 and 0.024, respectively), which means a positive or neutral variance is related to valgus or near normal NSA in the postoperative period and better functional outcomes. Shivashankar and Keshkar said likewise that one should aim at neutral with valgus or positive cortical reduction [9].

GTOL passed through the first quadrant in 14 (17.5%) subjects and through the second quadrant in 66 (82.5%) subjects. GTOL is significantly associated with NSA and functional outcome in our study with p values of 0.001 and 0011, respectively (Table 13). Antapur and Prakash found that the centre of the femoral head was 9.5 ± 6 mm below the tip of the GT in 82% of the cases [13]. They recommended using the tip of the GT as a reference for restoring the centre of the femoral head. Krishnan et al. found that the tip of the GT was at a higher level than the hip centre in 95% of the hips [14], and Theivendran et al. reported that the tip was 3.4 mm proximal to the centre of the femoral head [15]. In our study, we found a similar relationship between the tip of the GT and the centre of the femoral head; the tip was in the second quadrant in 82.5% (66 cases).

**Table 13:** Relation between GTOL and expected NSA GTOL: greater trochanter orthogonal line; NSA: neck shaft angle

GTOL	Expected NSA (in degrees)
First quadrant	<122
Second quadrant	>125

The most common position of the nail tip in the medullary canal is anterior in the lateral view (52.4%) and central in the AP view (58.75%). The anterolateral bow in the Indian/Asian population is most common as per a study done by Mahaisavariya et al. [16]. Therefore, there is difficulty in passing the nail beyond the isthmus and the need to ream 2 mm more than the nail diameter. There is a modification of the distal portion of the nail, which is tapered to pass through the isthmus. But inevitably because of the anterolateral bow, the nail hitches the anterior cortex and causes thigh pain in the follow-up (Table 14).

**Table 14:** Lateral thigh pain and position of nail tip AP: anteroposterior

Thigh pain (follow-up cases)	Position of nail tip in AP	Position of nail tip in lteral
8	Lateral	Anterior
3	Central	Anterior
1	Central	Central

**Figure 5:**
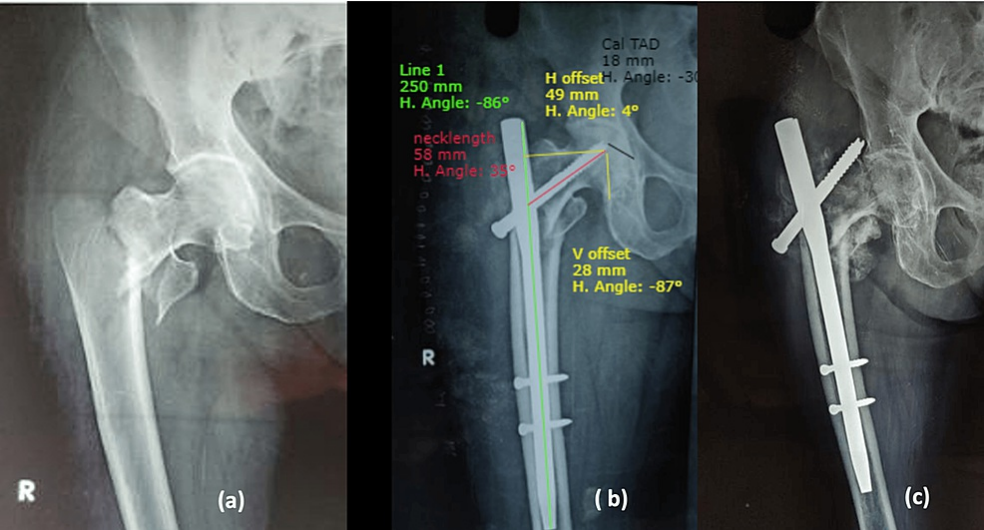
Reverse epsilon fracture with single screw (a) Reverse epsilon fracture; (b) Single screw fixation with vertical offset = 28 mm, horizontal offset = 49 mm, neck length = 58 mm, CalTAD = 18 mm, NSA = 120 degrees; (c) Screw cut out and varus collapse. H: horizontal; V: vertical; R: right; NSA: neck shaft angle; CalTAD: calcar referenced tip-apex distance

In one case due to improper reduction in varus and nail fixation, only one hip screw could be applied in superior part of femoral head, as there was no room available for the de-rotation screw. Eventually, screw back out and cut through happened with varus collapse in the follow-up (Figure 5). Appropriate reduction by releasing iliopsoas by open method and maintaining valgus reduction is the key as concurred by Chandak et al. [17]. Back-up plans of single helical blade screws or single screw systems would help in such situations.

**Figure 6:**
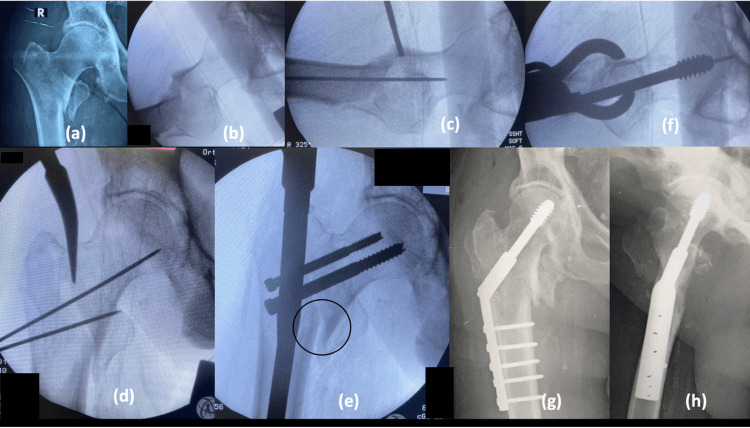
Intra-operative conversion from PFN to DHS (a and b) AP and lateral radiographs of unstable trochanteric fracture; (c) Percutaneous reduction and provisional fixation; (d) Entry through pyriformis; (e) Intertrochanteric translation after traction release; (f) Open reduction and DHS done; (g and h) Postoperative DHS fixation PFN: proximal femoral nail; DHS: dynamic hip screw; AP: anteroposterior

In four patients, the procedure was converted to DHS fixation due to severe GT comminution, nail passing through the fracture, lateralisation of nail, and coronal GT split (Figure 6). After traction is released, due to vertical fracture line, there is excessive sliding of proximal medial cortex leading to failure of reduction known as intertrochanteric translation. There are no documented studies that studied changes in the plan of surgery from PFN to DHS intraoperatively.

CRQC

Subjects were classiﬁed into poor fracture reduction (scores 0 and 1), acceptable fracture reduction (scores 2 and 3), and excellent fracture reduction (score 4). Among the 80 subjects, two had scores of 1, 11 had scores of 2, 39 had scores of 3, and 28 had scores of 4. Total fracture reduction quality was poor among two (2.5%) subjects, acceptable among 50 (62.5%) subjects, and excellent among 28 (35%) subjects. Since we did not compromise on fracture reduction by extending ourselves to use percutaneous or open methods if necessary, CRQC was fulfilled in the majority of cases including unstable fractures (Table 1). In our study, all the individual parameters are compared with CRQC for associations. In the study by Yan, et al [10] the quality of fracture reduction was good in 60 and 71, acceptable in 30 and 34, and poor in 6 and 7, respectively, in the GN group and PFNA group. These results are similar to that of our current study.

Postoperative parameters

The mean NSA reconstructed in stable fractures was 130.7±2.73 degrees and in unstable fractures was 128.9±3.44 degrees, which was almost equal to the NSA of the uninjured side, 131.0±3.14 degrees and 129.8±3.01 degrees, respectively (Table 4). This can be attributed to the good quality of reduction and placement of the screws in the head. The mean NSA lost after weight bearing among those with stable and unstable fractures was 2.0±1.17 degrees and 3.1±3.97 degrees, respectively, which is significant. In unstable fractures (postero-medial comminution), difficulty in reduction and low-grade Singh’s index in comparison to stable fractures lead to loss of NSA post weight bearing. Indicators of valgoid reduction, i.e. good NSA, are positive or neutral variance, Parker's ratio <0.33 in AP, and GTOL through the second quadrant (Table 15).

**Table 15:** Radiological parameters associated with NSA AP: anteroposterior; NSA: neck shaft angle; X^2^: Chi square

Radiological parameters	Χ^2^ value	p-value
Variance	15.071	0.005*
Parker’s ratio AP	17.864	<0.001*
Parker’s ratio Lateral	0.276	0.871
Greater Trochanter Orthogonal Line	30.072	<0.001*
Post-operative adverse events	61.870	<0.001*
*Level of significance: 0.05		

Neck length

**Figure 7:**
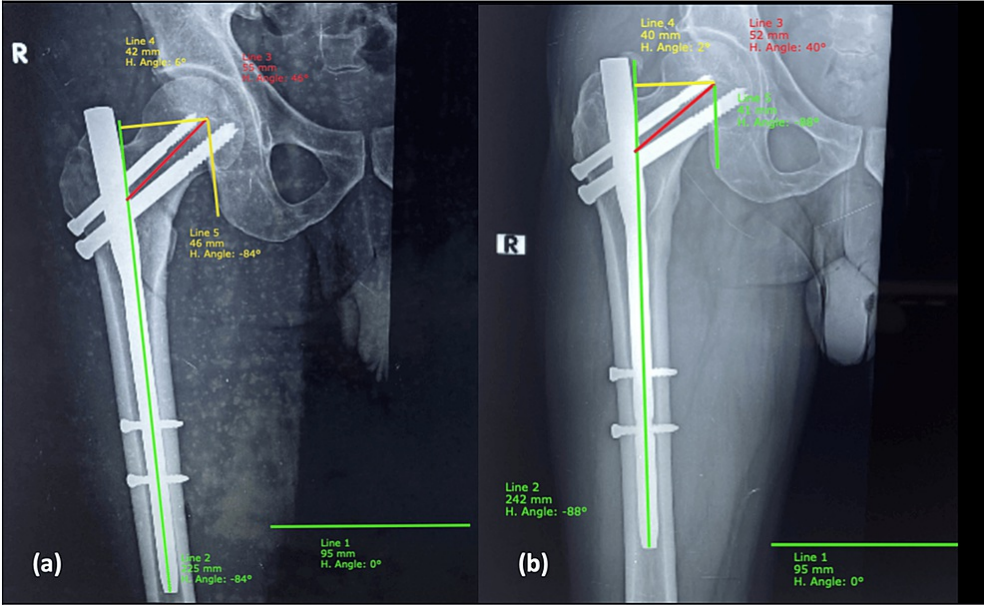
Neck length and horizontal offset (a) Immediate postoperative: Neck length (line 3) = 55 mm, Horizontal offset (line 4) = 42 mm; (b) Post weight bearing: Neck length (line 3) = 52 mm, Horizontal offset (line 4)= 40 mm H: horizontal; R: right

According to the current study, the mean neck length on the uninjured side among those with stable and unstable fractures was 59.2±3.57 mm and 57.6±3.64 mm, respectively. The mean neck length in the immediate postoperative period was 56.8±5.05 mm and 56.1±4.29 mm, respectively. The mean neck length lost after weight bearing (Figure 7) among those with stable and unstable fractures was 2.9±2.38 mm and 2.4±1.55 mm, respectively, with p-value of 0.261, which is not significant. In our study, we obtained an on-table compression of fracture by releasing traction before the completion of neck screw fixation; therefore, controlled collapse is not present and minimises the collapse of neck length in the follow-up after weight bearing. 

Horizontal offset

Mean reduction in the horizontal offset among those with stable and unstable fractures was 2.32±1.215 mm and 2.05±1.341 mm, respectively. The mean reduction in the horizontal offset among those with acceptable and excellent fracture reduction was 2.29±1.334 mm and 1.96±1.232 mm, respectively (Figure 7). The reduction was comparatively less among those with excellent fracture reduction and this was found to be statistically significant with a p-value of 0.036. The offset lost was more in unstable fractures with subnormal functional outcome, which is statistically significant with a p-value of 0.04. Gordon et al. found a strong correlation between loss of offset and reduction of hip function post weight bearing [18].

CalTAD

The mean CalTAD was 19.5±2.58 mm and 20.9±3.41 mm for stable and unstable fractures, respectively, with p-value of 0.08. Tip apex distance is probably not as important in PFN as it is in DHS as the TAD scale is linear and not multidirectional.

In the current study, the distribution of subjects was reported according to post-operative adverse events as there was abductor lurch in 11 subjects (13.7%), Z effect in one subject (1.5%), reverse Z effect in two subjects (2.5%), two subjects (2.5%) had screw cut out, and lateral thigh pain occurred in 12 subjects (15.0%), in one subject (1.3%) the implant was removed, and in one subject there was a peri-implant fracture. Implant-related mechanical complications occurred in 7/80 (8.7%).

The current study reported that among 80 subjects, according to HHS, three subjects (3.8%) had scores <70 with poor functional outcome, 29 subjects (36.2%) had scores of 80-90 with good functional outcome, and 48 subjects (60%) had scores of 90-100 with excellent functional outcome. 

The association between pre-op fracture status (both AO classification and Evans classification) and functional outcome was found to be statistically significant (p=0.031) meaning the majority of those with poor outcomes had unstable fractures while most of those with good and excellent outcomes had stable fractures. Also, the association between fracture reduction quality (CRQC) and functional outcome was found to be statistically significant (p<0.001) meaning majority of those with poor fractures reduction had poor outcome while most of those with good and excellent outcome had excellent/acceptable fracture reduction.

Subjects with good fracture reduction quality (p<0.001), entry point medial to GT tip or piriformis, NSA > 128 degrees (p<0.001), no intraoperative adverse events (p<0.001), continuous anterior cortical alignment (p<0.001), positive or neutral variance (p=0.024), Parker’s ratio <0.33 (p=0.001), position of Calcar screw inferior/central in AP, and GTOL passing through the second quadrant (p=0.011) had more excellent functional outcomes. Other variables were also evaluated but did not play the role of significant factors associated with good functional outcomes.

Limitations

Limitations included the loss of subjects in the follow-up period through deaths. This study is about the parameters for good reduction and outcomes and there was no comparison with newer devices like PFNA and trochanteric femoral nail. 

## Conclusions

Trochanteric fractures in elderly osteoporotic individuals need early optimization and proper surgery for early mobilization which reduces complications, as mobility is one of the most significant qualities of life. Though PFN is technically challenging, with proper technique, it gives excellent results. The most important technical aspects are achieving good non-varus reduction, inserting the nail correctly, and accurate placement of lag screws. The technique of provisional fixation of fracture fragments by Steinmann-pin/K-wires significantly helps in all four major steps of PFN which include reduction, entry, nail passing, and lag screw fixation. Aiming for biomechanically stable reduction either by closed or open means is the key to treating unstable intertrochanteric fractures.

Unstable fractures should be initially reduced to a slightly valgus position, or near normal NSA because the NSA decreases after weight bearing. One should never accept varus reduction. A good entry point leads to proper nail and screw placement; therefore, the entry point should always be medial to the tip or piriformis according to the fracture pattern. Tip apex distance should be kept to a minimum, its AP component, i.e. the lag screw, especially should be inserted deeply into the femoral head, close to subchondral bone kissing the calcar. Tip apex distance is probably not as important in PFN as it is in DHS, as the TAD scale is linear and not multidirectional.

## Appendices

**Table 16:** Questionnaire CR: Commercial Registration; No: Number; BMI: Body Mass Index; H/O: History Of; AO/OTA: Arbeitsgemeinschaft für Osteosynthesefragen/Orthopaedic Trauma Association; NSA: Neck Shaft Angle; CalTAD: Calcar Tip Apex Distance; AP: Anteroposterior; Lat: Lateral; GTOL : Greater Trochanter Orthogonal Line

Name :	
Age :	
Sex :	
Height :	
BMI:	
CR No :	
Address :	
Phone no :	
Date of Injury:	
Date of presentation:	
Date of Surgery:	
Total duration of stay:	
Mode of injury:	
Side of injury:	
Associated injuries:	
Comorbidities if any:	
History of previous medications (if any):	
Level of physical activity:	
Ambulation status :	
Previous H/O similar injury or osteoporotic fracture in opposite hip :	
Investigations :	
Bedsore :	
Singh’s Index :	
Metabolic bone disease/Bone pathology (if any) :	
Diagnosis :	
Intertrochanteric fracture classification :	
Boyd and Griffin class:	
Evans class:	
AO/OTA Class:	
Surgical details	
Interval between injury and surgery :	
Interval between presentation and surgery :	
Parameters taken into consideration	
1)Reduction Manoeuvre Acceptable or not	Closed / Percutaneous / Open
2) Entry point :	
3) Anterior cortical reduction :	
4) Variance:	
5) NSA-Immediate post op :	
6) NSA of follow up after weight bearing :	
7) NSA loss after weight bearing :	
8) Loss of Neck length after weight bearing on follow up:	
9) The hip screw placement: In Antero posterior view :	
10) The hip screw placement : In Lateral view :	
11) Parker’s ratio: Anteroposterior : Lateral :	
12) CalTAD : (AP calTAD) + (lat) =	
13) Length of the nail :	
14) Diameter of the nail :	
15) Length of lag screw :	
16) Horizontal offset (immediate post op):	
17) Horizontal offset (post weight bearing):	
18) Vertical offset (immediate post op) :	
19) Vertical offset (post weight bearing) :	
20 Length of de-rotation screw:	
21) Distal locking :	
22) Chang’s quality of fracture reduction :	
23) GTOL :	
24) Anterior Cortical Line :	
25) Amount of blood loss :	
26) Operating time:	
27) Post op Blood transfusion : NIL	
28)Adverse events:	
Intraoperative:	
Immediate:	
Late:	
29) Harris hip score at 6 months :	
30) Gait evaluation at 6 months :	
31) Abductor strength :	

## References

[1] Panula J, Pihlajamäki H, Mattila VM, Jaatinen P, Vahlberg T, Aarnio P, Kivelä SL (2011). Mortality and cause of death in hip fracture patients aged 65 or older: a population-based study. BMC Musculoskelet Disord.

[2] Jonnes C, Shishir SM, Najimudeen S (2016). Type II intertrochanteric fractures: proximal femoral nailing (PFN) versus dynamic hip screw (DHS). Arch Bone Jt Surg.

[3] Murena L, Moretti A, Meo F, Saggioro E, Barbati G, Ratti C, Canton G (2018). Predictors of cut-out after cephalomedullary nail fixation of pertrochanteric fractures: a retrospective study of 813 patients. Arch Orthop Trauma Surg.

[4] Mao W, Ni H, Li L, He Y, Chen X, Tang H, Dong Y (2019). Comparison of Baumgaertner and Chang reduction quality criteria for the assessment of trochanteric fractures. Bone Joint Res.

[5] Mahomed NN, Arndt DC, McGrory BJ, Harris WH (2001). The Harris hip score: comparison of patient self-report with surgeon assessment. J Arthroplasty.

[6] Singh M, Nagrath AR, Maini PS (1970). Changes in trabecular pattern of the upper end of the femur as an index of osteoporosis. J Bone Joint Surg Am.

[7] Akan K, Cift H, Ozkan K, Eceviz E, Tasyikan L, Eren A (2011). Effect of osteoporosis on clinical outcomes in intertrochanteric hip fractures treated with a proximal femoral nail. J Int Med Res.

[8] Jain MJ, Mavani KJ, Patel D (2017). Role of provisional fixation of fracture fragments by Steinmann-pin and technical tips in proximal femoral nailing for intertrochanteric fracture. J Clin Diagn Res.

[9] Shivashankar B, Keshkar S (2021). Intertrochanteric fractures: ten commandments for how to get good results with proximal femoral nailing. Indian J Orthop.

[10] Yan D, Soon Y, Lv Y (2012). Proximal femoral nail antirotation versus Gamma nail in treatment of femoral trochanteric fractures. Curr Orthop Pract.

[11] Menezes DF, Gamulin A, Noesberger B (2005). Is the proximal femoral nail a suitable implant for treatment of all trochanteric fractures?. Clin Orthop Relat Res.

[12] Morihara T, Arai Y, Tokugawa S, Fujita S, Chatani K, Kubo T (2007). Proximal femoral nail for treatment of trochanteric femoral fractures. J Orthop Surg (Hong Kong).

[13] Antapur P, Prakash D (2006). Proximal femoral geometry: a radiological assessment. J Arthroplasty.

[14] Krishnan SP, Carrington RW, Mohiyaddin S, Garlick N (2006). Common misconceptions of normal hip joint relations on pelvic radiographs. J Arthroplasty.

[15] Theivendran K, Hart WJ (2009). Is the tip of the greater trochanter a reliable reference for the rotation centre of the femoral head in total hip arthroplasty?. Acta Orthop Belg.

[16] Mahaisavariya B, Sitthiseripratip K, Oris P, Chaichanasiri E, Suwanprateeb J (2004). Fit-and-fill analysis of trochanteric gamma nail for the Thai proximal femur: a virtual simulation study. J Med Assoc Thai.

[17] Chandak R, Malewar N, Jangle A, Agarwal R, Sharma M, Kekatpure A (2019). Description of new "epsilon sign" and its significance in reduction in highly unstable variant of intertrochanteric fracture. Eur J Orthop Surg Traumatol.

[18] Gordon M, Berntsson PO, Sjölund E, Demir Y, Hedbeck CJ, Stark A, Sköldenberg O (2016). Loss of offset after pertrochanteric hip fractures affects hip function one year after surgery with a short intramedullary nail. A prospective cohort study. Int Orthop.

